# Bone lesions of the tibia: Multimodal iconographic review and diagnostic algorithms, Part 2: Metaphyseal and epiphyseal lesions

**DOI:** 10.1016/j.ejro.2025.100654

**Published:** 2025-05-01

**Authors:** Vincent Salmon, Pedro Augusto Gondim Teixeira, Alain Blum

**Affiliations:** Service d’Imagerie Guilloz, Hôpital Central, CHRU Nancy, Nancy 54000, France

**Keywords:** Tibia, Bone lesions, Metaphysis, Epiphysis

## Abstract

This article focuses on the analysis of bone lesions of the tibia, addressing the main diagnostic challenges and imaging strategies used to characterize them. It examines the different etiologies of tibial lesions, emphasizing the importance of a systematic approach to distinguishing tumoral from non-tumoral lesions, as well as from bone dysplasia. The article underlines the essential role of imaging, particularly radiography, CT, and MRI, in accurate lesion characterization. It also highlights typical clinical and radiological features that help guide diagnosis and management. The main aim is to provide radiologists with clear guidelines for improving the identification of bony lesions of the tibia. Part 2 of this 2-part article proposes some illustrations of metaphyseal and epiphyseal lesions of the tibia.

## Highlights

• Multimodal imaging enhances diagnosis of tibial metaphyseal and epiphyseal lesions.
• Systematic imaging helps distinguish tumoral and non-tumoral bone lesions of the tibia.
• Clinical and imaging features helps diagnostic approach of tibial bone lesions.

## 1 Introduction

In the second part of this 2-part review of bone lesions of the tibia, we develop some metaphyseal and epiphyseal lesions. They can present significant challenges for diagnosis. This review primarily focuses on the multimodal imaging characteristics of benign and malignant tibial lesions.

## 2 Metaphyseal lesions

### 2.1 Cortical defect, non-ossifying fibroma

Cortical defect and non-ossifying fibroma are benign fibrous tumors with identical histology. Their size and symptomatic nature differentiate them: cortical defect refers to a lesion less than 20 mm in size and asymptomatic, while non-ossifying fibroma refers to a lesion greater than 20 mm in size or symptomatic (pain, pathological fracture).

These lesions are among the most common benign bone tumors, particularly in children and adolescents, affecting 25–50 % of cases, with a male-to-female ratio of 2:1. They are very rare in newborns and adults. In most cases, they regress spontaneously in adulthood.

These lesions may form part of the Jaffe-Campanacci syndrome, in which case they are numerous and associated with extra-skeletal manifestations (cutaneous, ocular, cardiovascular, genital, intellectual impairment, etc.).

Cortical defects and non-ossifying fibromas are typically located in the metaphyseal region, often on the posteromedial aspect of the cortical bone. They may migrate toward the diaphysis as the individual grows. Their development is thought to be linked to the traction of tendons on the bone cortex [1].

**Fig. 1:**
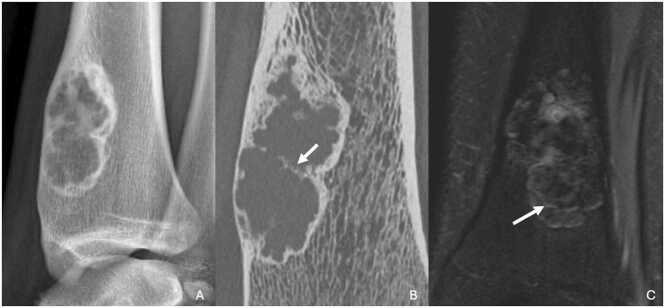
Cortical defect of the left tibia in a 19-year-old young man incidentally discovered in extension study of SAMS bacteremia. A. Profile radiography of the left tibia showing oval radiolucent lesion with sclerotic margins centered on the cortex of the tibial distal metaphysis. B, CT image in the sagittal plane highlighting this osteolytic lesion with sclerotic margins, with some internal septa (white arrow). C. T2-weighted fat-suppressed MR image in the coronal plane showing a heterogenous signal of the lesion, mainly in T2 hyposignal with internal septa in T2 hypersignal (white arrow).

Diagnosis is usually made incidentally on standard radiographs that reveal a well-limited, oval osteolytic lesion with sclerotic margins (Fig. 1). The long axis of the lesion is parallel to that of the supporting bone. The cortical bone may be slightly blown but remains continuous. Internal septa may be present.

If a CT scan is performed due to diagnostic uncertainty or atypical features, similar characteristics are observed. The lesion appears osteolytic, with sclerotic contours.

On MRI, the lesion is typically T1 hyposignal and T2 hyposignal, although it may sometimes show a T2 hypersignal. Margins and internal septa are often T2 hypersignal, with occasional enhancement after gadolinium injection [2]. There is no associated soft-tissue abnormality.

Bone scintigraphy shows weak to moderate hyperfixation.

### 2.2 Fibrous dysplasia

Fibrous dysplasia is an uncommon benign bone disorder, accounting for 5–7 % of benign bone tumors. However, it is considered more of a developmental anomaly rather than a true neoplasm, potentially caused by a mutation in the GNAS gene [3], [4].

The condition can present in either a monostotic form, affecting a single bone, or a polyostotic form, involving multiple bones. The monostotic form is the most common, representing 75 % of cases, and is often discovered incidentally in adolescents and young adults under 30. It can also be responsible for pain and pathological fractures. Polyostotic fibrous dysplasia may be associated with syndromes such as McCune-Albright syndrome (involving café-au-lait spots and endocrinopathies) or Mazabraud syndrome (featuring intramuscular myxomas). The type of GNAS mutation could also be associated with the monostotic or polyostotic form of fibrous dysplasia [5].

Tibial involvement can result in deformity and curvature. In long bones such as the tibia, fibrous dysplasia typically localizes to the metaphysis or diaphysis, often with centromedullary involvement.

**Fig. 2:**
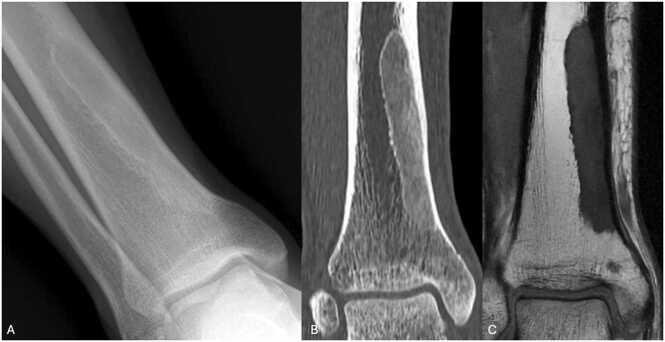
Fibrous dysplasia in the distal third of right tibia in a 32-year-old man presenting pain. A. Face radiography showing a large oval ground-glass lesion with sclerotic margins of the distal third of right tibia. B, CT image in the coronal plane highlighting the aspect of ground-glass. C. T1-weighted MR image in the coronal plane showing a homogenous hyposignal with hyposignal corona (white arrow).

On standard radiographs, the lesion usually appears osteolytic, with a characteristic ground-glass appearance and sclerotic margins (Fig. 2). Boundaries are sharp. Cortical bone may be blown away but remains continuous. More ossified lesions may present with increased osteocondensation.

Characteristics of fibrous dysplasia on CT scan are similar, with a central ground-glass area, more or less dense depending on ossification, and with sclerotic margins.

On MRI, lesion is hyposignal T1. T2 signal of the central zone is variable [6]. A peripheral T1 and T2 hyposignal corona is characteristic. Cystic or hemorrhagic changes may be present. Signal abnormalities and adjacent soft tissue contrast may be present in up to one-third of cases.

Bone scintigraphy usually reveals hyperfixation, which can be helpful in detecting additional lesions in polyostotic forms.

### 2.3 Chondroma

Chondroma is a common benign cartilage tumor, second only to osteochondroma in prevalence [7]. It accounts for 10–25 % of benign bone tumors and is typically discovered in adolescents and young adults aged 10–40. When multiple, these tumors may be associated with Ollier disease or Maffucci syndrome, both of which carry an elevated risk of malignant transformation into chondrosarcoma [8], [9].

Chondroma can be either medullary (called central chondroma or enchondroma) or periosteal (peripheral chondroma, periosteal, juxta-cortical or ecchondroma). They most commonly affect the small bones of the hands, but they can also occur in long bones. The distal metaphysis of the tibia is particularly affected, where lesions usually measure less than 5 cm. This tumor is often discovered incidentally but may cause pain or pathological fractures.

On standard radiographs, a chondroma is a metaphyseal or metadiaphyseal, osteolytic lesion, centered or eccentric. Its margins are lobulated and geographic, with no peripheral sclerosis. Popcorn, arch or ring calcifications are characteristic of cartilaginous lesions. Cortical bone may appear blown, but for less than two-thirds of its thickness.

**Fig. 3:**
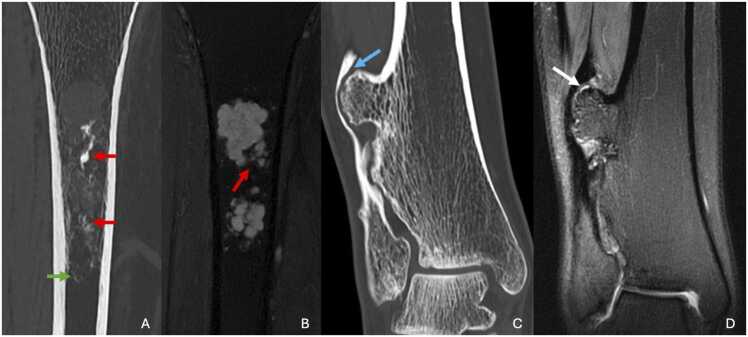
A,B. Enchondroma of the proximal third of the right tibia in a 50-year-old woman. C,D. Osteochondroma of the distal metaphysis of the right tibia in a 20-year-old woman (different patient). A. CT image in the coronal plane showing an osteomedullar osteolytic lesion with central popcorn calcifications (red arrow) and thin arc calcifications (green arrow), without endosteal scalloping. B. T2-weighted fat-suppressed MR image in the coronal plane showing a multilocuted lesion with cartilaginous matrix: hypersignal with some marked hyposignal corresponding to calcifications (red arrow). C. CT image in the coronal plane showing a pedunculated bony outgrowth presenting continuity with supporting bone. Notice the mass effect on the fibula (blue arrow) D. T2-weighted fat-suppressed MR image in the coronal plane showing a thin cartilaginous cap (white arrow).

CT scans can more precisely define calcifications and endosteal scalloping (Fig. 3).

On MRI, the chondromal matrix produces a distinct T2 hypersignal and T1 hyposignal, with non-signal areas corresponding to calcifications (Fig. 3). Medial septa, if present, exhibit T1 and T2 hyposignal. The lesion typically demonstrates strong arc-like or ring-shaped contrast enhancement at the periphery and septa. In rare cases of large, blown chondroma, adjacent soft-tissue involvement may be seen.

Bone scintigraphy shows hyperfixation, which can be useful in identifying additional chondromas in cases of multiple lesions.

Criteria for suspecting chondrosarcoma degeneration are: pain, epiphyseal localization, size greater than five cm and growth in size, endosteal scalloping greater than two-thirds of cortical thickness, disappearance of calcifications, periosteal reaction, soft tissue involvement, superior fixation to the anterior superior iliac spine on scintigraphy [10].

### 2.4 Osteochondroma

Osteochondroma, also known as exostosis, is the most common benign bone tumor, accounting for 20–50 % of all benign bone tumors [11]. It may occur as a solitary lesion or as part of hereditary multiple exostoses. Osteochondromas have a predilection for the metaphysis or the metaphyseal-diaphyseal junction of long bones, particularly in the lower limbs. The proximal metaphysis of the tibia is affected in 15–20 % of cases.

This lesion is very often asymptomatic and discovered incidentally, usually before the age of 20. Sex ratio is balanced. Osteochondroma may cause swelling or deformity of the limb. Neurovascular compression is possible, as surface bursitis associated with adjacent soft-tissue irritation [12]. Arterial or venous thrombosis in contact with the osteochondroma is possible [13]. In case of pain, sarcomatous degeneration should also be suspected, which is slightly more frequent in hereditary multiple exostoses (3–5 % of cases versus 1 % in the case of solitary tumors).

On standard radiographs, osteochondroma appears as a bony outgrowth, continuous with the cortex and medulla of the supporting bone. It may be large. Two forms can be distinguished: sessile, corresponding to a broad implantation base, or pedunculated, with a narrower stalk. Importantly, the lesion always points away from the metaphysis towards the diaphysis.

CT scans better demonstrate the cortico-medullary continuity between the lesion and the supporting bone (Fig. 3). They can reveal irregularities in the cartilaginous cap, which often contains calcifications. Changes in calcifications over time, particularly a reduction in the number of calcifications, are suggestive of malignant transformation.

MRI is particularly useful for evaluating the cartilaginous cap, especially in cases of suspected sarcomatous transformation. The cap exhibits T2 hypersignal and T1 hyposignal. Its thickness decreases with age, measuring subcentimetric in adults. In children and adolescents, it can measure up to 20–30 mm. Malignant transformation is suspected if the thickness exceeds 15–20 mm in adults or 30 mm in children [12]. Medial septa and periphery of the lesion may enhance after gadolinium injection, but this is not suggestive of malignancy. MRI is also effective at identifying soft tissue compression, such as bursitis, caused by the lesion. Pes anserinus syndrome or medial hamstring snapping knee syndrome have been described [14], [15].

Bone scintigraphy shows hyperfixation in the cartilaginous cap, which diminishes with age.

### 2.5 Chondromyxoid fibroma

Chondromyxoid fibroma is a very rare benign cartilage tumor, representing less than 1 % of primary bone tumors. It most commonly affects young adults between the ages of 20 and 30, with a slight male predominance [7]. This tumor has a topographical preference for the lower limbs, particularly the proximal tibia. Its typical location is metaphyseal, which helps distinguish it from chondroblastoma. Chondromyxoid fibroma is often asymptomatic, but some patients may experience localized, progressive pain.

On standard radiographs, the lesion appears as an eccentrically located osteolytic mass within the medullary cavity, aligned along the long axis of the tibia. The margins are well-defined, lobulated, and often surrounded by a thin rim of peripheral sclerosis. The lesion may cause cortical thinning or even rupture, although periosteal reactions are rare [16]. Internal trabeculations are frequently observed. Unlike other cartilage tumors, calcifications are rarely seen.

**Fig. 4:**
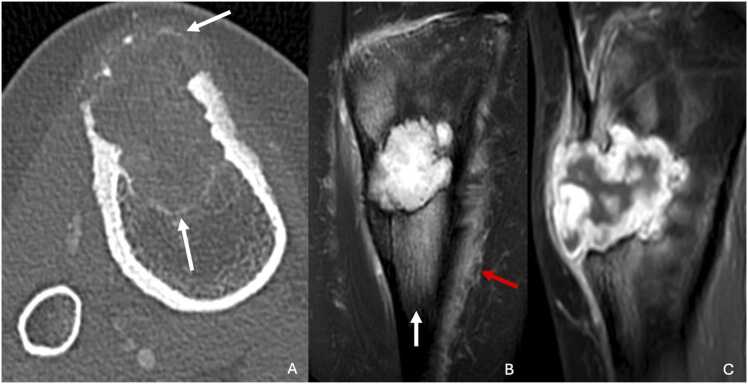
Chondromyxoid fibroma in a 19-year-old woman with painless swelling of the proximal third of the right leg for two years. A. CT image in the axial plan showing a cortical disruption of this eccentric osteomedullar lesion, with thin sclerosis margins (white arrows). B. Proton density-weighted fat-suppressed MR image in the coronal plane showing intense hypersignal with adjacent osteomedullar edema(white arrow) and soft tissue edema (red arrow). C. T1-weighted fat-suppressed contrast-enhanced MR image in the sagittal plane showing mainly peripheral enhancement without enhancement of central myxoid areas.

CT scans provide better delineation of the cortical integrity and allow assessment of soft tissue extension (Fig. 4).

On MRI, chondromyxoid fibroma shows T1 hyposignal and either iso- or hypersignal on T2-weighted images. The sclerotic margins and trabeculations within the lesion are T1 and T2 hypointense. Enhancement after gadolinium administration is typically heterogeneous, with no contrast enhancement in the myxoid portions. Although soft tissue extension is rare, MRI is excellent for detecting such involvement if present.

Bone scintigraphy usually shows moderate hyperfixation at the lesion's periphery.

### 2.6 Essential bone cyst

An essential bone cyst, also known as a simple bone cyst, is an uncommon benign tumor containing fluid. It accounts for approximately 3 % of all bone tumors. It is typically seen in children and young adults, with rare cases after the age of 30. Boys are affected more frequently, at a ratio of 3:1 compared to girls [17]. The cyst is usually asymptomatic, though pathological fractures can occur.

The proximal metaphysis of long bones is the most common location. In adults, the lesion may migrate toward the diaphysis as the individual grows. The cyst is typically centromedullary, with its long axis aligned with that of the bone.

On standard radiographs, the lesion is a unilocular osteolytic area with a thin peripheral sclerotic border, giving an "egg-cup" appearance. The “fallen fragment sign,” which occurs when a bone fragment falls into the cyst after a pathological fracture, is pathognomonic of an essential bone cyst.

CT scans show the cyst's fluid-like density, confirming the diagnosis.

On MRI, the cyst typically exhibits a classic fluid signal, although liquid-liquid levels may occasionally be seen. Peripheral shell contrast enhancement is possible.

Bone scintigraphy is generally unhelpful, as there is no increased uptake [18].

### 2.7 Aneurysmal bone cyst

An aneurysmal bone cyst (ABC) is a rare benign bone tumor with fluid content, accounting for approximately 1 % of primary bone tumors. ABCs can be diagnosed at any age but are more commonly seen in patients younger than 20 [19]. Pain and swelling are the most common presenting symptoms.

Aneurysmal bone cysts can be classified as primary, representing 70 % of cases, or secondary, arising in association with other bone tumors such as giant cell tumors or chondroblastomas. The lesion is typically located in the metaphysis of long bones like the tibia. It is eccentric to the bone’s long axis.

On standard radiographs, ABCs appear as well-circumscribed, multilocular osteolytic lesions with internal septa [20]. The cortical bone may be expanded but remains intact.

CT scans confirm these findings, revealing well-demarcated cortical boundaries and multiple internal lobulations, whose density varies depending on hemorrhagic changes. CT imaging also demonstrates the natural evolution of the lesion, with progressive ossification of the septa.

**Fig. 5:**
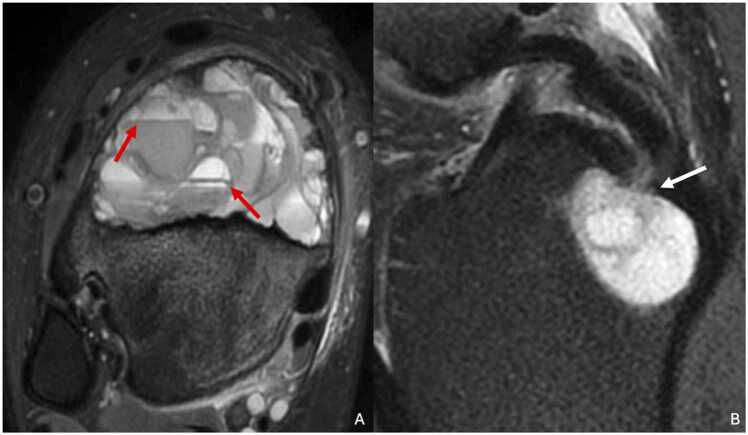
A. Aneurysmal bone cyst of the distal metaphysis of the right tibia in a 15-year-old male adolescent with pain persistent after a sprain. B. Intraosseous mucoid cyst of the proximal epiphysis of the right tibia in a 43-year-old man (different patient). A. T2-weighted fat-suppressed MR image in the axial plane showing a large heterogenous lesion with liquid-liquid levels (red arrows). B. T2-weighted fat-suppressed MR image in the sagittal plane showing a 2 cm cystic lesion of the proximal epiphysis communicating with the insertion of the posterior cruciate ligament (white arrow).

MRI is useful for identifying heterogeneous T1 and T2 signals and the presence of liquid-liquid levels within the lesion [21], [22] (Fig. 5). Liquid-liquid levels correspond to sedimentation of blood components. They are suggestive of an aneurysmal bone cyst but are not specific. The internal septa show a fibrous signal, appearing hypointense on both T1- and T2-weighted images.

Bone scintigraphy shows hyperfixation, but this finding is non-specific.

The main differential diagnoses include giant cell tumors and telangiectatic osteosarcomas, both of which can present with similar imaging features, including fluid-fluid levels.

### 2.8 Giant cell tumor

Giant cell tumor (GCT) is an uncommon benign bone tumor with locally aggressive potential. It accounts for approximately 5 % of primary bone tumors and typically affects adults between the ages of 20 and 50 [23]. While it is more common in women, aggressive forms of GCT are more frequently seen in men (sex ratio 3:1).

In some cases, this benign tumor may metastasize, particularly to the lungs, resulting in so-called "benign" pulmonary metastases. Rare forms of GCT may become malignant, either de novo or secondarily, often following radiotherapy. The clinical course is variable, with the tumor sometimes discovered following a pathological fracture causing pain. Recurrence is not uncommon despite surgical excision, particularly if there are pulmonary metastases [24].

GCTs preferentially affect the long bones, particularly the proximal metaphysis of the tibia, and often extend into the epiphysis [25]. The lesion is typically eccentric, with a cortico-medullary location, and may involve the subchondral bone.

On standard radiographs, it appears as a blowing osteolytic lesion, generally large (over 5 cm). Margins are well-defined and there is sometimes thin peripheral osteosclerosis.

**Fig. 6:**
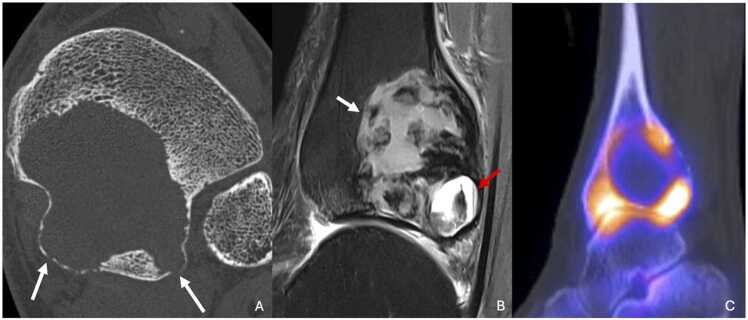
Giant cell tumor in a 59-year-old man with mechanically induced left ankle pain. A. CT image in the axial plane highlighting a large osteolytic lesion with multifocal cortical disruptions (white arrows). B. T2-weighted fat-suppressed MR image in the sagittal plane showing heterogenous signal with solid (white arrow) and cystic areas (red arrow). C. Bone scintigraphy in the sagittal plane highlighting “the donut sign” corresponding to peripheral hyperfixation.

CT scans can provide better evaluation of the cortical integrity and are especially useful when ultra-high-resolution (UHR) imaging is available. CT can also confirm the absence of internal calcifications (Fig. 6).

On MRI, the lesion typically shows weak to intermediate signal on both T1 and T2-weighted images, although areas of increased T2 signal may occasionally be present. After gadolinium injection, strong and early enhancement is observed in perfusion sequence [26]. A secondary aneurysmal bone cyst is often associated with GCTs, dividing the tumor into solid and cystic areas with liquid-liquid levels. MRI is also excellent for assessing adjacent soft tissue involvement.

Bone scintigraphy frequently demonstrates a characteristic “donut sign,” with intense peripheral uptake and reduced activity in the central portion (Fig. 6).

### 2.9 Osteosarcoma

Osteosarcoma is rare but it is the second most common primary malignant bone tumor, following multiple myeloma. Conventional osteosarcoma represents approximately 75 % of cases, with rarer subtypes such as telangiectatic and periosteal osteosarcoma also occurring [27], [28]. Males are more frequently affected, with most patients being between 10 and 25 years of age [29]. Secondary osteosarcoma can develop later in life, typically after the age of 40, in individuals with risk factors such as Paget's disease or prior radiation therapy.

In most cases, osteosarcoma affects the metaphysis of long bones. The proximal metaphysis of the tibia is a frequent site. The tumor usually begins intramedullary and rapidly extends to the cortex and surrounding soft tissues. Pain is the primary symptom, with swelling and limited range of motion following. Pathological fractures are possible but uncommon.

Osteosarcoma is often suspected based on standard radiographs, which reveals a mixed sclerotic and lytic lesion with indistinct margins and invasion of the surrounding soft tissues [30]. Aggressive features are typically present, including cortical osteolysis (Lodwick type 2 or 3), soft tissue invasion, and a multilamellar periosteal reaction (e.g., "sunburst" appearance or Codman’s triangle).

CT scans better characterize these findings, although standard radiographs are often sufficient for diagnosis. CT is particularly useful for planning treatment and assessing the tumor's extent, especially for identifying pulmonary metastases.

**Fig. 7:**
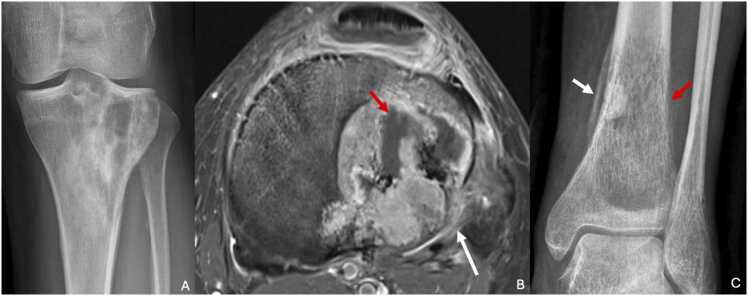
A,B. Osteosarcoma in a 42-year-old woman with left gonalgia for six months. C. Ewing sarcoma of the left tibia in a 45-year-old woman incidentally discovered after a trauma (different patient). A. Face radiography showing a heterogenous lesion of the proximal third of left tibia with blurred boundaries. B. T1-weighted fat-suppressed contrast-enhanced MR image in the axial plane showing an intense enhancement of this tumor with necrotic central areas (red arrow). Notice adjacent osteomedullar edema, periosteal reaction and extraosseous extension (white arrow). C. Face radiography showing a large osteolytic lesion with lytic cortex (red arrow) and plurilamellar periosteal reaction (white arrow).

MRI typically reveals T1 hypointensity and T2 hyperintensity, with significant contrast enhancement (Fig. 7). This imaging modality is ideal for assessing soft tissue invasion and detecting skip metastases within the same bone or extension to the epiphysis and adjacent joints [31]. MRI should include the joints above and below the lesion.

Bone scintigraphy and ^18^FDG-PET scans are useful for identifying skip metastases and distant metastases.

### 2.10 Ewing sarcoma

Ewing sarcoma is a rare bone tumor but represents the second most common primary malignant bone tumor in children after osteosarcoma. Most patients are under 30 years old, with a slight male predominance [32].

The tumor primarily affects long bones, particularly at the metaphyseal-diaphyseal junction, as well as the pelvis and ribs. Pain is the predominant symptom, often accompanied by swelling and, in some cases, general systemic symptoms. Pathological fractures are rare, and the tumor is usually localized at diagnosis.

Ewing sarcoma appears as an osteolytic lesion originating in the medulla, with extension toward the cortex. The cortex appears lysed (Lodwick type 2 or 3), and the lesion often extends into the surrounding soft tissues. A periosteal reaction, classically described as “onion skin” or spiculated, is common [33] (Fig. 7). Calcifications are rare, which helps differentiate Ewing sarcoma from osteosarcoma.

CT scans provide better visualization of cortical destruction, periosteal reactions, and soft tissue extension. A thoracic CT scan is essential for evaluating pulmonary metastases, which are common with this tumor (25 % of cases at diagnosis) [34].

MRI is valuable for assessing the local extent of the disease, including the medullary involvement and the search for skip metastases (less common than in osteosarcoma), as well as soft tissue extension. On T1-weighted images, Ewing sarcoma appears hypointense, and it is usually hyperintense on T2-weighted images. Large lesions may present heterogeneous T2 signals due to necrosis or hemorrhage. Perilesional bone marrow edema is common. Post-gadolinium images show marked enhancement, which helps delineate tumor boundaries from surrounding edema. Multilamellar periosteal reactions appear as T2 hypointense striations.

Bone scintigraphy shows non-specific tumor hyperfixation, while ^18^FDG-PET scan is recommended for staging to detect metastases [35].

Main differential diagnoses consist of osteosarcoma, osteomyelitis and non-Hogdkin lymphoma. Those lesions can be difficult to distinguish in imaging. Percutaneous biopsy is often needed to obtain the right diagnosis [34], [36].

### 2.11 Bone lymphoma

Primary bone lymphoma is a rare form of bone cancer, accounting for less than 5 % of all primary malignant bone tumors. It is typically a large B-cell non-Hodgkin lymphoma, though Hodgkin lymphoma can also involve the bone. This cancer affects individuals of all ages but is most commonly diagnosed in patients between 45 and 60 years old, with a slight male predominance [37], [38]. Disseminated lymphoma with bone involvement is more frequent than primary bone lymphoma.

Long bones, such as the tibia, are most affected (in 70 % of cases), with a preference for the metaphyseal-diaphyseal junction. At diagnosis, bone lymphoma is often limited to the bone or involves regional lymph nodes, with distant lymphatic spread occurring later. About 25 % of cases present with a pathological fracture. Bone pain and swelling are common, though systemic symptoms are typically mild.

On standard radiographs, appearance is variable, but bone lymphoma most often corresponds to an osteolytic lesion with Lodwick type 2 or 3 cortical involvement. In some cases, the lesion is mixed, but it is rarely osteocondensing. Aggressive periosteal reactions are seen in more than 50 % of cases [34].

CT scans provide a better view of cortical destruction and soft tissue involvement.

MRI typically shows the tumor as T1 hypointense and T2 hyperintense, with homogeneous contrast enhancement. Intense soft tissue infiltration is a hallmark of bone lymphoma and is well-visualized with MRI [39].

^18^FDG PET scans are particularly useful for detecting distant metastases and for assessing the full extent of disease involvement.

### 2.12 Hemangioma

Bone hemangioma is a benign vascular tumor that is common in the spine but rare in long bones. When hemangiomas affect the tibia, they are typically found at the metadiaphyseal junction within the medullary cavity. Rarely, they can be in the cortex or periosteum. Most spinal hemangiomas are asymptomatic, but hemangiomas in the appendicular skeleton are often associated with pain [40].

On standard radiographs, hemangioma is a well-demarcated radiolucent lesion, with coarse trabeculae [41]. Cortical thinning or expansion may also occur.

**Fig. 8:**
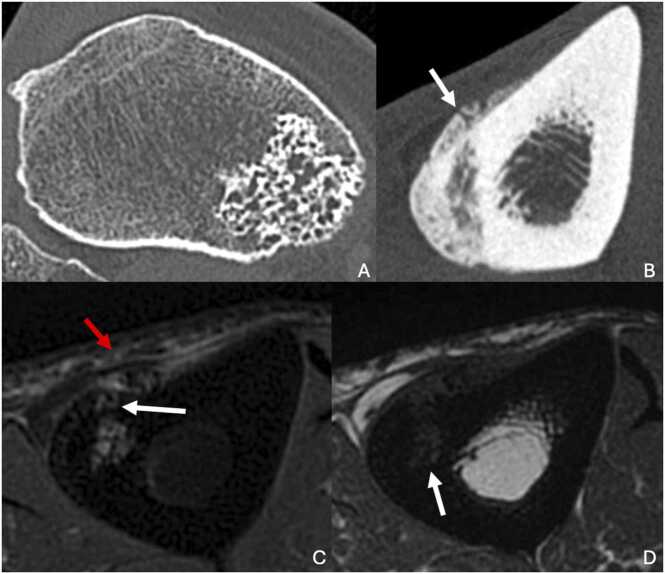
A. Corticomedullar hemangioma of the proximal third of the right tiba in a 53-year-old man presenting medial right knee pain. B,C,D. Cortical hemangioma of the middle third of the left tibia, proven after surgical biopsy, in a 59-year-old man (different patient). A. CT image in the axial plane showing a honeycomb appearance of an eccentric osteomedullar lesion. B. CT image in the axial plane showing cortical thickening with periosteal reaction (white arrow). C. T2-weighted fat-suppressed MR image in the axial plane showing multiple hypersignal aeras separated by hyposignal internal septas (white arrow), with a microvessel inside the cortical thickening (white arrow). Notice the periosteal reaction and soft-tissue adjacent edema (red arrow). D. T1-weighted MR image in the axial plane showing presence of fat in this lesion (white arrow).

CT scans are more specific, revealing classic features such as the "polka-dot sign" (due to thickened trabeculae around intralesional vessels), osteolysis, or a honeycomb pattern with multiple thin internal trabeculations (Fig. 8). In some cases, a spiculated "Irish lace" appearance may be seen [42]. It may also present an aggressive appearance with cortical destruction.

On MRI, the lesion typically shows non-specific T1 hypointensity and T2 hyperintensity, with contrast enhancement. There may be portions of T1 and T2 hypersignal, representing fat within the lesion. The lesion’s margins and internal trabeculations are hypointense on both T1- and T2-weighted sequences [42].

Bone scintigraphy is usually negative unless the lesion is aggressive, in which case there may be increased uptake.

### 2.13 Osteomyelitis and osteitis

Osteomyelitis is an inflammation of spongious bone, common in pediatric population, and is mostly due to infection.

It mainly affects metaphyseal bones of lower limbs, particularly the tibia in 48–73 % of cases [43]. Annual incidence is one case per 5000 children. Boys are more often affected. There are two peaks in incidence: in children under five and in adults over 50. In children, hematogenous spread is the most common cause of infection, while direct inoculation, typically due to open fractures, is more common in adults. Methicillin-resistant *Staphylococcus aureus* (MRSA) is a frequent causative organism. The growth plate acts as a barrier in children over 18 months, protecting the epiphysis from infection.

Clinical presentation includes fever, pain, erythema, and swelling of the affected limb. Occasionally, these symptoms are absent, making diagnosis more difficult.

**Fig. 9:**
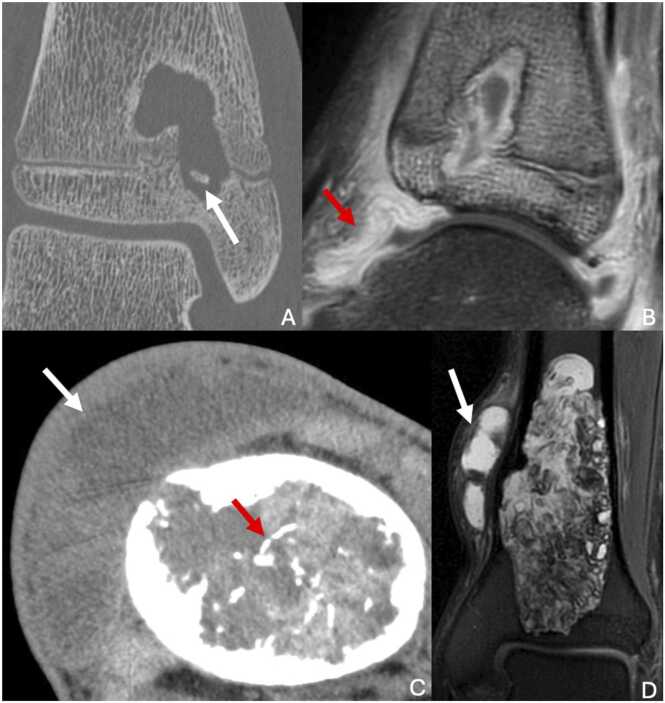
A,B. Osteomyelitis with Brodie’s abcess of the distal metaphysis of right tibia in a 16-year-old male adolescent presenting with inflammatory pain in the right ankle for three months, with a biological inflammatory syndrome. C,D. Bone hydatid cyst of the distal third of left tibia in a 43-year-old man from Mali with 18 months of left leg pain (different patient). A. CT image in the coronal plane showing an osteolytic lesion with bone sequestration (white arrow), centered in the metaphysis. B. T1-weighted fat-suppressed contrast-enhanced MR image in the sagittal plane showing peripheral enhancement of the Brodie’s abcess, adjacent osteomedullar enhancement in relation to bone edema and an important synovitis tibiotalar joint (red arrow). C. CT image in soft tissue reconstruction in the axial plane showing heterogenous content of an osteolytic lesion, with thin central linear calcifications (red arrow), and extension to soft tissue (white arrow). D. T2-weighted fat-suppressed MR image in the coronal plane highlighting multiple daughter vesicles inside the large bone vesicle associated with pluriloculated soft-tissue collection (white arrow).

Osteomyelitis progresses through two stages: an acute stage lasting less than two weeks and a chronic stage if infection persists. During the acute stage, standard radiographs may show periosteal reactions and soft tissue swelling in fewer than 20 % of cases [44]. Bone rarefaction and poorly defined radiolucent images may also be present, raising suspicion of malignant pathology. Subacute osteomyelitis is characterized by a well-demarcated oval osteolytic lesion known as Brodie's abscess (Fig. 9). In chronic osteomyelitis, bone sequestration is visible on standard radiographs, presenting as an oval sclerotic mass surrounded by a radiolucent ring, with cortical thickening and trabecular disorganization [45].

CT scans help detect bone sequestration and periosteal reactions.

MRI is the imaging modality of choice for early diagnosis. It can detect osteomedullary edema as T1 hypointensity and T2 hyperintensity with contrast enhancement. Periosteal detachment and soft tissue abscesses can also be visualized. Brodie’s abscesses show a characteristic target-like appearance on MRI, with a liquid center surrounded by a T1 and T2 hyperintense penumbra. The "double-line sign" has also been described, corresponding to a T2 hypersignal surrounded by a T2 hyposignal border [44]. In chronic stage, bone sequestrations are hyposignal on T1- and T2-weighted sequences and do not enhance after gadolinium injection.

Bone scintigraphy is sensitive for detecting osteomyelitis early, showing increased radiotracer uptake before radiographic changes become apparent, although it lacks specificity [46]. Unfortunately, this hyperfixation is aspecific and MRI is more discriminating in acute stage. In chronic infection, ^18^FDG-PET scanning is useful for detecting signs of ongoing infection [47].

Differential diagnosis with Ewing sarcoma can be challenging. Classic symptoms of infection may be absent in children. Aggressive features on imaging, such as periosteal reaction and soft tissue invasion, can lead to misdiagnosis. Sharp and defined margin of the bone lesion is highly suggestive of Ewing sarcoma [48].

Osteitis refers to infection confined to the cortical bone. It can occur as part of SAPHO syndrome (Synovitis, Acne, Pustulosis, Hyperostosis, Osteitis) or its pediatric counterpart, chronic recurrent multifocal osteomyelitis (CRMO). These conditions may have an infectious, autoimmune, or genetic etiology. SAPHO syndrome mainly affects young adult population, while CRMO mainly concerns children and adolescents [49]. As the acronym SAPHO indicates, these pathologies encompass osteoarticular, cutaneous and digestive manifestations.

Spine, pelvis and sternoclavicular region are classic sites of osteoarticular reach and are generally responsible for pain or limitation of joint amplitude. Metaphysis of long bones, such as the tibia, are also frequently affected by CRMO. Diaphyseal or proximal metadiaphyseal topography of the tibia is more common in SAPHO syndrome, and accounts for only 30 % of bone involvement.

On imaging, osteitis shares many features with osteomyelitis, including osteolysis, cortical thickening, and trabecular disorganization. However, in chronic osteitis, bone sequestration is absent. MRI reveals significant osteomedullary edema but no abscesses or sequestration [50].

### 2.14 Bone hydatid cyst

Tibia can be the site of atypical infectious pathologies, such as hydatidosis. Bone involvement occurs in only 0.5–2.5 % of cases of hydatidosis but is considered one of the most severe forms of the disease [51]. It is caused by *Echinococcus granulosus*. Humans are intermediate hosts, after ingestion of food or water contaminated with animal feces containing parasite eggs.

Bone involvement in this infection predominates in richly vascularized areas [52]. It first affects spine, then long bones (femur and tibia in decreasing order of frequency). Infection is initially centromedullary in metaphysis, spreading to epiphysis and later to diaphysis.

Bone hydatid disease is typically asymptomatic for an extended period. By the time it is detected, the damage is often extensive, and patients may present with pathological fractures, limb deformities, or superinfection. Hypereosinophilia may be seen in some cases.

On standard radiographs, the disease manifests as multiple osteolytic lesions (lacunae) that respect the general morphology of the bone. It is important to note that there is no periosteal reaction or peripheral sclerosis. Intraosseous calcifications are uncommon, but when present, they are characteristic. In rare cases, hydatidosis can mimic chronic osteomyelitis with areas of osteolysis, peripheral sclerosis, periosteal reaction, and soft tissue extension, or it may resemble a pseudotumoral lesion with bone expansion [53].

CT scans are more informative, showing multiloculated osteolytic lesions with fluid density and the absence of contrast enhancement. Calcifications within soft-tissue collections are also well visualized with CT (Fig. 9).

MRI is particularly useful in diagnosing hydatid disease, showing characteristic fluid signal intensity within the cysts. "Daughter vesicles" inside a larger "mother vesicle" are pathognomonic and help differentiate this disease from other conditions [52].

Bone scintigraphy shows non-specific hyperfixation [54].

### 2.15 Erdheim-Chester disease

Erdheim-Chester disease (ECD) is a very rare form of non-Langerhansian histiocytosis, with around 800 cases reported worldwide [55]. Since 2016, it has been recognized as a neoplastic pathology linked to recurrent mutation of MAPK activation pathway [56], [57]. It can occur at any age, but preferentially affects adults, with a mean age at diagnosis of 56 years and an M:F sex ratio of 2.6:1 [58].

Bone involvement occurs in 95 % of cases, but there is also an extra-osseous involvement (retroperitoneal, particularly renal with the appearance of hairy kidneys, cardiovascular, genital, pulmonary, endocrine, neurological, cutaneous, etc.). This pathology is responsible for an altered general condition. Bone pain is present in almost 50 % of cases, of moderate but permanent intensity, and predominantly in the lower limbs. Bone involvement of peripheral skeleton has a quasi-pathognomonic presentation. The tibia is affected in nearly 75 % of cases, followed by the femur in 66 % [59]. Distribution is bilateral and symmetrical, with a preferential diaphyseal localization, followed by a metaphyseal localization.

**Fig. 10:**
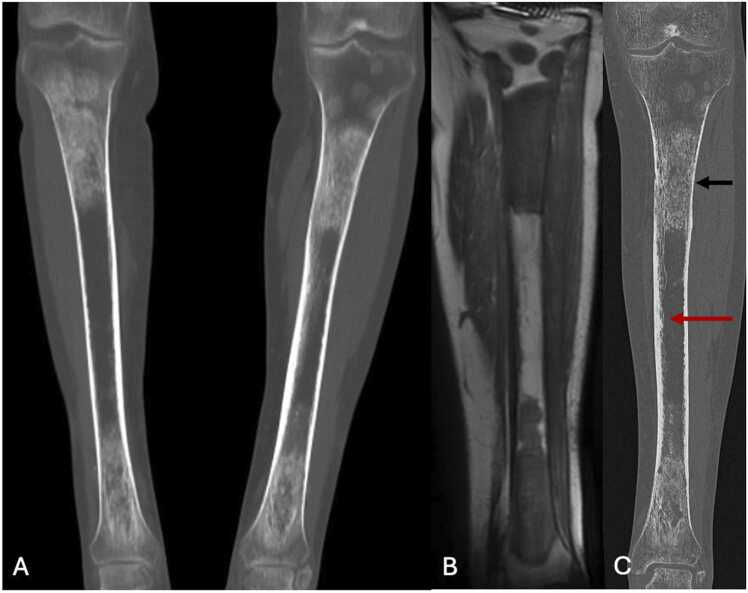
Erdheim-Chester disease in a 50-year-old man. A. Reformation in coronal plane of bone scintigraphy showing bilateral and symetric osteosclerosis of proximal and distal metadiaphyseal junctions of tibias. B. T1-weighted MR image of the left tibia in coronal plane showing marked hyposignal of those sclerotic areas. C. UHR CT of the left tibia in coronal plane highlighting those sclerotic areas of the medulla (black arrow) with better visualization of endosteal involvement of cortical thickening in the middle diaphysis (red arrow).

On standard radiographs, ECD appears as diffuse medullary osteosclerosis of the diaphysis and metaphysis, with associated cortical thickening (Fig. 10). Occasionally, radiolucent lesions within the osteocondensed bone may be observed, along with cortical erosion and soft tissue invasion.

CT scans provide a detailed assessment of cortical and medullary involvement and help evaluate the extent of multivisceral involvement, which is critical for prognosis.

MRI clearly shows osteomedullary replacement with heterogeneous intermediate T2 hypersignal and heterogeneous T1 hyposignal (signal inferior to that of muscle). Enhancement is also heterogeneous. Periostitis may be present.

Bone scintigraphy and ^18^FDG-PET scanning show hyperfixation of both osseous and extra-osseous lesions, often preceding radiographic changes.

### 2.16 Brown tumor

Despite its name, brown tumor is not really a tumor. It is a fibrous tissue consisting of an accumulation of osteoclastic giant cells around hemorrhagic foci. It is a rare benign osteolytic process, occurring in 3 % of cases of primary hyperparathyroidism and between 1.5 % and 1.7 % of cases of secondary hyperparathyroidism. In forms secondary to chronic renal failure, women over 50 are slightly more affected than men.

The metaphysis of long bones, including the tibia, is a common site for brown tumors. Bone involvement may be solitary or multifocal, with bone pain or pathological fractures being the main clinical symptoms. Lesions may be asymptomatic.

On standard radiographs, brown tumor is an eccentric, osteolytic lesion with well-defined contours [60]. Cortical thinning and, occasionally, cortical rupture may be observed, but there is no periosteal reaction. As the lesion evolves, internal septa may develop, giving the lesion a lobulated appearance.

CT scans can highlight cortical rupture and the internal septations of the tumor, which have a ground-glass density [61].

MRI typically shows T1 hypointensity or isointensity and T2 hyperintensity, with gadolinium enhancement of the solid portions and septations. Liquid-liquid levels may be present due to hemorrhagic phenomena within the cystic cavities [62]. There is no invasion of adjacent soft tissue.

Bone scintigraphy shows intense hyperfixation, but this finding is non-specific.

Treatment of the underlying hyperparathyroidism usually leads to tumor regression, with the lesion being replaced by osteosclerotic tissue.

### 2.17 Bone infarct

Bone infarct corresponds to aseptic osteonecrosis of a mineralized area of bone due to a lack of blood supply. It has many causes, including trauma, long-term corticosteroid use, alcohol abuse, systemic diseases (sickle cell disease, systemic lupus erythematosus, Gaucher's disease) [63]. Bone infarcts are not very rare. They commonly occur at the metaphyseal-diaphyseal junction but may also affect subchondral epiphyseal bone.

More than 50 % of bone infarcts are solitary, but multiple infarcts can occur in the context of systemic diseases. Bone infarcts are generally asymptomatic unless associated with a severe ischemic crisis, as seen in sickle cell disease.

**Fig. 11:**
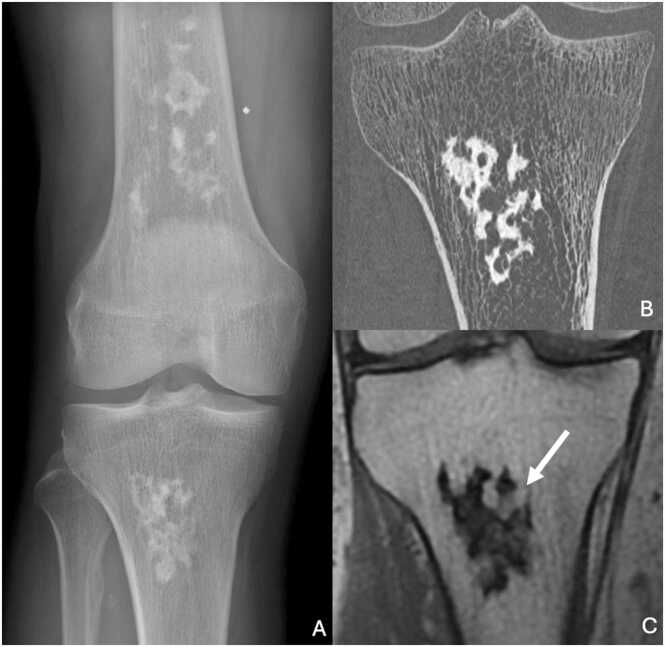
Bone infarcts of right femur and tibia in a 41-year-old man. A. Face radiography showing serpiginous sclerotic femoral and tibial lesions, producing a “smoke volute” appearance. B. UHR CT image in the coronal plane higlighting the serpiginous sclerotic ostemedullar lesion of proximal tibia. C. T1-weighted Dixon (in phase) MR image in the coronal plane showing a marked hyposignal of the lesion corresponding to sclerotic portions and some fatty areas with signal similar to adjacent normal osteomedullar bone (white arrow).

On standard radiography, early infarcts appear as radiolucent or osteocondensed areas with non-specific findings. Established infarcts show a characteristic serpiginous sclerotic contour, producing a “smoke volute” appearance [64] (Fig. 11). There is no periosteal reaction. The main differential diagnosis is chondroma.

CT scans help distinguish between bone infarcts and chondromas, with the latter often showing central calcifications.

On MRI, early infarcts appear as poorly circumscribed areas of T2 hyperintensity, which progressively increase in size and delineate well-defined contours. Chronic infarcts present as T1 hypointense lesions with the classic “double rim sign” on T2-weighted sequences: a central area of sclerosis surrounded by repair tissue in T2 hyperintensity [65]. The central necrotic region does not enhance, while the periphery shows contrast enhancement. In chronic stage, the lesion center shows a variable signal, very often fatty, similar to that of perilesional bone marrow. Sometimes, the lesion center is liquid or fibrous, always surrounded by a border.

Bone scintigraphy typically reveals hypoactivity in early infarcts, with normalization or hyperfixation in later stages.

Rarely, infarcts may degenerate into sarcomas. Pain combined with osteolysis may raise suspicion of malignancy.

### 2.18 Osgood-Schlatter disease

Osgood-Schlatter disease is an osteochondrosis of the anterior tibial tuberosity (ATT), resulting from partial avulsion due to repetitive microtrauma. This condition commonly affects adolescent males between 11 and 15 years of age, particularly those engaged in sports requiring sudden knee extension, such as soccer or jumping. One-third of cases are bilateral [66]. The secondary ossification center of the ATT appears between ages 8 and 12 in girls and between 9 and 14 in boys. Diagnosis is typically clinical, with anterior knee pain exacerbated by activity and relieved by rest. The pain can be reproduced by extending the knee against resistance, and there may be swelling over the anterior knee. X-rays are performed to rule out fractures, infection, or tumors in atypical presentations. Cases of periosteal chondroma, osteochondroma and hemimelic epiphyseal dysplasia have been reported [67].

Standard radiographs show soft tissue swelling over the ATT and a blurred appearance of the distal insertion of patellar tendon. If secondary ossification nucleus is not yet present, ossifications opposite the ATT appear after one month of painful evolution. If secondary ossification nucleus is already present, it appears fragmented and avulsed. Tibial cortical irregularities may be present.

Ultrasound demonstrates hypoechogenicity of the fragmented ossification center and the distal third of the patellar tendon, along with associated Doppler hyperemia.

**Fig. 12:**
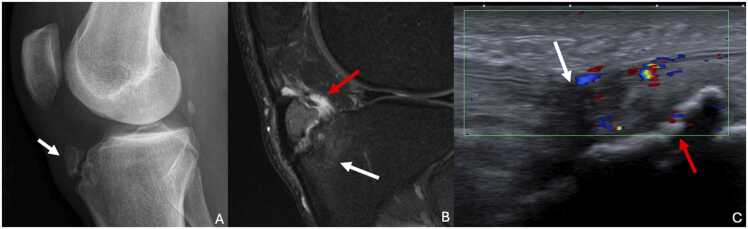
A,B. Osgood-Schlatter disease of the left tibia in a 37-year-old man presenting with pain and swelling of the anterior aspect of the left knee, with no notion of trauma. C. Osgood-Schaltter disease of the left tibia in a 11-year-old boy (different patient). A. Profile radiography showing avulsion of secondary ossification nucleus of the anterior tibial tuberosity (ATT) (white arrow). Corticalisation of margins is in favour of an old avulsion. B. T2-weighted fat-suppressed MR image in the sagittal plane showing bone edema of this secondary ossification nucleus and ATT (white arrow) with inflammatory signal of adjacent soft-tissue, more specifically infrapatellar fat pad (red arrow). C. Doppler ultrasound image in the sagittal plane showing irregularities of ATT (red arrow) with hypoechogenicity (white arrow) and hyperhemia of the distal enthesis of patellar tendon.

MRI reveals T2 hyperintensity and T1 hypointensity centered on the ATT, with an avulsed appearance of the ossification center [68]. Inflammatory changes in adjacent soft tissues are common (Fig. 12).

Ultrasound and MRI can also be used to search for associated infrapatellar bursopathy. CT scan and bone scintigraphy are performed in exceptional cases.

## 3 EPIPHYSEAL LESIONS

### 3.1 Intraosseous mucoid cyst

Intraosseous mucoid cysts, also known as juxta-articular bone cysts, are relatively common benign lesions that can occur at any age, with a slight male predominance. These cysts are typically unilocular, juxta-articular, and located in the cancellous bone, frequently involving the proximal tibia. Distal tibial involvement is rarer [69]. Unlike subchondral cysts, intraosseous mucoid cysts are not associated with frank degenerative joint changes.

These cysts may cause pain but are often discovered incidentally.

On standard radiography, intraosseous mucoid cysts appear as well-defined osteolytic lesions, usually between 1 and 2 cm in size, with a thin sclerotic border [70]. No periosteal reaction is observed.

CT scans confirm the radiographic findings, and an arthrography may reveal communication with the adjacent joint.

On MRI, the cyst shows T1 hypointensity or isointensity and T2 hyperintensity (Fig. 5). Fluid levels and adjacent bone marrow edema may be present [70], [71]. Thin peripheral contrast enhancement may be present.

Bone scintigraphy demonstrastes aspecific hyperfixation.

### 3.2 Gout

Gout is a microcrystalline disease caused by the deposition of monosodium urate crystals, resulting from chronic hyperuricemia. It is a common condition, affecting approximately 1 % of the population. It is four to ten times more prevalent in men. Its incidence increases with age.

Clinically, gout typically presents as recurrent episodes of acute arthritis. Chronic tophaceous gout is the advanced, progressive form of the disease and is characterized by subcutaneous tophi, para-articular bone erosions, and bone proliferation. Intraosseous tophi can also develop, forming within the bone cortex near joints and giving a pseudotumoral appearance.

On standard radiographs, intraosseous tophus appears as an oval, well-limited lacuna with sclerotic borders, oriented along the diaphyseal axis. It may mimic a subchondral macro-geode when close to a joint. Tophus may be solitary or multiple. Intra-lesional calcifications are sometimes present [72].

**Fig. 13:**
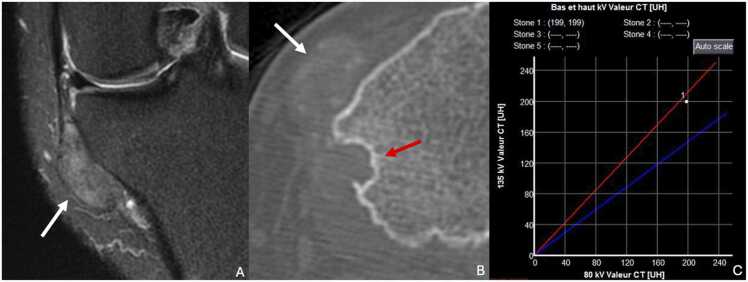
Gout in a 53-year-old man with pain and swelling on the medial side of the left knee for six months. A. T2-weighted fat-suppressed MR image in the coronal plane showing a well-contoured tophus para-articular of the left knee in moderate hypersignal (white arrow). B. CT image in the axial plane showing the tophus with discrete calcifications (white arrow) and scalopping of the tibial cortex (red arrow). C. Dual-energy CT confirms presence of monosodium urate crystals and the diagnostic of gouty tophi.

CT scans offer a more detailed evaluation of cortical involvement and the relationship of the lesion with adjacent joints. Dual-energy CT scans are particularly useful for identifying monosodium urate crystals with greater specificity [73] (Fig. 13).

On MRI, gouty tophi are well-contoured and exhibit moderate T1 hypointensity and T2 hyperintensity. Contrast enhancement is primarily peripheral. Bone edema in the surrounding area is generally mild.

^18^FDG PET scan shows hypermetabolism in gouty tophi, but this finding is non-specific [74].

### 3.3 Chondroblastoma

Chondroblastoma, also known as Codman’s tumor, is a rare benign cartilage tumor, accounting for less than 1 % of bone tumors. It predominantly affects adolescents and young adults under the age of 25, typically before the closure of the growth plate. It is two to three times more common in males. In the tibia, the proximal extremity is most frequently involved.

Clinically, chondroblastoma presents with knee pain, joint stiffness, and, occasionally, joint effusion.

On standard radiographs, chondroblastoma presents as a round or oval, well-limited or map-shaped osteolytic lesion [75]. Margins are sclerotic in almost 60 % of cases. The lesion is eccentric to the epiphysis, often in contact with the growth plate. It measures between 1 and 5 cm in diameter. The cortex may be thinned or blown out, with associated periosteal apposition. Calcifications are visible in fewer than half of the cases on X-rays.

CT scans provide better visualization of internal calcifications and trabeculations, as well as the lesion’s relationship with the growth plate and cortical bone. Liquid-liquid levels may be present, particularly when an aneurysmal bone cyst is associated.

**Fig. 14:**
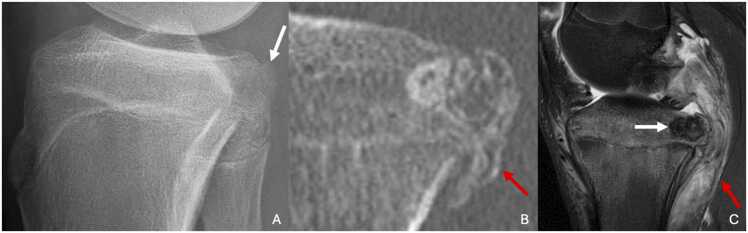
Chondroblastoma in 15-year-old male adolescent with post-traumatic right gonalgia. A. Profile radiography showing a radiolucency on the posterior part of the tibial plateau (white arrow). B. CT image in the sagittal plane highlighting an osteolytic lesion with sclerotic margins of the proximal epiphysis of tibia associated with periosteal reaction (red arrow). C. T2-weighted fat-suppressed MR image in the sagittal plane showing a pronounced hyposignal of the lesion, characteristic of chondroblastoma (white arrow), surrounding by bone marrow edema and intense adjacent soft-tissue inflammatory reaction (red arrow).

On MRI, the lesion is hyposignal T1 and variable T2 signal, often hyposignal T2, which is characteristic of this cartilaginous tumor [76] (Fig. 14). The sclerotic margins show T1 and T2 hypointensity. There is often edema of the adjacent bone marrow. Liquid-liquid levels are observed if an associated aneurysmal bone cyst is present. Contrast enhancement is heterogeneous and moderate. MRI also provides superior assessment of joint involvement, including reactive synovitis.

On bone scintigraphy, there is an intense tumor hyperfixation.

Because of its epiphyseal location, the main differential diagnosis is clear-cell chondrosarcoma (CCCS) [75], [77]. However, CCCS occurs in older patients. It is often larger and presents T2 hypersignal. Adjacent bone oedema is less pronounced in CCCS.

### 3.4 Osteopathia striata

Osteopathia striata, also known as Voorhoeve disease, is a rare benign bone dysplasia that affects individuals of all ages and genders. It can be inherited in an autosomal dominant manner or occur sporadically.

The condition primarily affects fast-growing long bones, such as the tibia, particularly at the epiphysis and metaphysis [78]. It corresponds to a failure of mature cortical bone remodeling. Although it can affect any bone, the skull and clavicles are generally spared. In rare cases, it has been associated with skull sclerosis or focal dermal hypoplasia, which is seen in Goltz-Gorlin syndrome [79], [80].

Osteopathia striata is often bilateral but can sometimes be unilateral. The condition is typically asymptomatic and is most often discovered incidentally during imaging.

On standard radiographs, the diagnosis is usually confirmed by the presence of dense, linear, thin, and uniform striations oriented along the diaphyseal axis of the bone [81].

There is no hyperfixation on bone scintigraphy [82].

## 4 Conclusion

This review has explored a wide spectrum of bone lesions affecting the tibia, ranging from benign conditions to malignant tumors. Although some of these lesions share common clinical and radiographic features, a systematic approach that combines clinical context with advanced imaging techniques is essential for accurate diagnosis and management. This narrative review briefly describes the main bone lesions to help orientating the right diagnosis in imaging.

Multimodal imaging modalities such as radiography, CT, MRI, and bone scintigraphy play crucial roles in the detection, characterization, and management of these lesions. A comprehensive understanding of the clinical presentation and radiological features is essential for accurate diagnosis and appropriate patient management.

New advanced imaging modalities under development, such as PET-MRI, could possibly help characterize bone lesions. The development of AI tools to assist radiology may also soon be part of the diagnostic aids.

## Funding Statement

This work did not receive any specific grant from funding agencies in the public, commercial, or not-for-profit sectors.

## Author contributions

All authors attest that they meet the current International Committee of Medical Journal Editors (ICMJE) criteria for Authorship.

## Human and animal rights

Not applicable.

## Informed consent and patient details

The authors declare that this report does not contain any personal information that could lead to the identification of the patient(s).

## CRediT authorship contribution statement

**Blum Alain:** Visualization, Validation, Supervision, Conceptualization. **Gondim Teixeira Pedro Augusto:** Visualization, Validation. **Salmon Vincent:** Writing – review & editing, Writing – original draft, Validation, Formal analysis.

## Declaration of Generative AI and AI-assisted technologies in the writing process

During the preparation of this work the authors used ChatGPT in order to improve language and readability. After using this tool/service, the authors reviewed and edited the content as needed and take full responsibility for the content of the publication.

## Declaration of Competing Interest

The authors declare that they have no known competing financial interests or personal relationships that could have appeared to influence the work reported in this paper.
